# Assessment of Tilapia Skin Collagen for Biomedical Research Applications in Comparison with Mammalian Collagen

**DOI:** 10.3390/molecules29020402

**Published:** 2024-01-13

**Authors:** Jyun-Yuan Huang, Tzyy-Yue Wong, Ting-Yuan Tu, Ming-Jer Tang, Hsi-Hui Lin, Yuan-Yu Hsueh

**Affiliations:** 1 International Center for Wound Repair and Regeneration, National Cheng Kung University, Tainan City 701, Taiwan; yyuangtw@gmail.com (J.-Y.H.); 10806045@gs.ncku.edu.tw (T.-Y.W.); tingyuan@mail.ncku.edu.tw (T.-Y.T.); mjtang1@mail.ncku.edu.tw (M.-J.T.); 2 Department of Biomedical Engineering, College of Engineering, National Cheng Kung University, Tainan City 701, Taiwan; 3 Medical Device Innovation Center, National Cheng Kung University, Tainan City 701, Taiwan; 4 Department of Physiology, College of Medicine, National Cheng Kung University, Tainan City 701, Taiwan; 5 Division of Plastic and Reconstructive Surgery, Department of Surgery, National Cheng Kung University Hospital, College of Medicine, National Cheng Kung University, Tainan City 701, Taiwan; 6 Center of Cell Therapy, National Cheng Kung University Hospital, College of Medicine, National Cheng Kung University, Tainan City 701, Taiwan

**Keywords:** tilapia skin collagen, fibril formation, cell migration, cyst formation, gel contraction, interface invasion, wound healing

## Abstract

Collagen is an important material for biomedical research, but using mammalian tissue-derived collagen carries the risk of zoonotic disease transmission. Marine organisms, such as farmed tilapia, have emerged as a safe alternative source of collagen for biomedical research. However, the tilapia collagen products for biomedical research are rare, and their biological functions remain largely unexamined. In this study, we characterized a commercial tilapia skin collagen using SDS-PAGE and fibril formation assays and evaluated its effects on skin fibroblast adhesion, proliferation, and migration, comparing it with commercial collagen from rat tails, porcine skin, and bovine skin. The results showed that tilapia skin collagen is a type I collagen, similar to rat tail collagen, and has a faster fibril formation rate and better-promoting effects on cell migration than porcine and bovine skin collagen. We also confirmed its application in a 3D culture for kidney cells’ spherical cyst formation, fibroblast-induced gel contraction, and tumor spheroid interfacial invasion. Furthermore, we demonstrated that the freeze-dried tilapia skin collagen scaffold improved wound closure in a mouse excisional wound model, similar to commercial porcine or bovine collagen wound dressings. In conclusion, tilapia skin collagen is an ideal biomaterial for biomedical research.

## 1. Introduction

Collagen is the most abundant animal protein, making up 25% to 35% of the body’s total protein content. Due to its excellent cell binding properties, biocompatibility, low antigenicity, and high biodegradability, collagen has been widely used as a biomaterial in various two- or three-dimensional cell culture experiments and in fabricating scaffolds that mimic native tissue [1]. Collagen is primarily extracted from mammalian skin and tendon tissues, such as from pigs, bovines, and rat tails. However, concerns over zoonotic disease transmission and religious restrictions have led researchers to seek alternative, safer sources of collagen, such as marine organisms like fish, sponges, and jellyfish [2].

Over 70% of the fish caught is processed in factories, generating significant amounts of fish waste, including heads, fins, viscera, scales, bones, and skin. The disposal and recycling of fish waste is a key issue in terms of economic and environmental concerns. Due to its high collagen content, fish waste has been extensively extracted for use in the food, pharmaceutical, and cosmetic industries [3]. Fish collagen has also been suggested as a biomaterial for biomedical applications. However, concerns have been raised about its lower levels of proline and hydroxyproline content compared to mammalian collagen since proline and hydroxyproline contain imino rings and form hydrogen bonds to confer collagen mechanical strength and conformation stability [4,5]. However, collagen derived from warm water fish species, such as tilapia, which has higher proline and hydroxyproline content (~25%) than cold water fish (~17%), could exhibit similar properties in terms of thermostability and rigidity to that of collagen from mammalian sources, such as porcine and bovine (~30%) [6,7].Tilapia is one of the most farmed fish globally [8], making it an ideal resource for developing tilapia collagen biomedical products due to its abundant resource and low cost. Reports have shown that tilapia collagen can modulate immune cell activity [9], induce osteogenic differentiation [10,11], and promote wound healing [12,13]. However, collagen products derived from tilapia for biomedical research are rare, and their biological functions require further exploration.

In this study, we evaluated the efficacy of commercial tilapia skin collagen in cell experiments by comparing it with other sources of type I collagen, including rat tail, porcine skin, and bovine skin. We examined the effects of these collagens on the cell adhesion, proliferation, and migration of a human skin fibroblast cell line. We also investigated the application of the tilapia skin collagen on 3D cell culture models. Additionally, we freeze-dried the tilapia skin collagen solution to form a scaffold and applied it to full-thickness skin wounds in mice to evaluate its effect on wound healing. Our results indicate that tilapia skin collagen is an ideal biomaterial for biomedical research.

## 2. Results

### 2.1. The Composition and Fibril Formation of the Tilapia Skin Collagen

**Figure 1:**
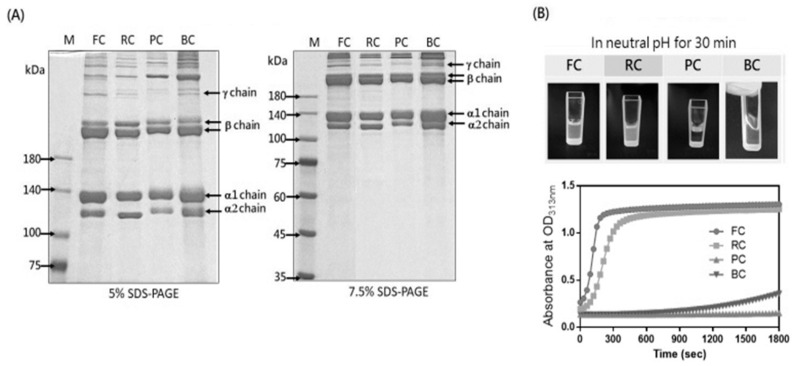
SDS-PAGE analysis and fibril formation of the collagens. (**A**) SDS-PAGE analysis of tilapia skin collagen (FC), rat tail collagen (RC), porcine skin collagen (PC), and bovine skin collagen (BC) was performedusing5% or 7.5% SDS-PAGE. Following staining with Coomassie Blue R 250, the collagen bands corresponding to theα1, α2, β, andγ chains were observed. (**B**) To initiate self-assembly, the collagen samples were treated with H_2_O and 10× PBS to achieve a neutral solution with a 1 mg/mL concentration. The kinetics of fibril formation in the collagen samples were assessed by measuring the optical density (OD) at 313 nm over 30 min using a spectrophotometer.

We characterized the tilapia skin collagen (FC) by comparing it with other commercial type I collagens, namely a rat tail collagen (RC), porcine skin collagen (PC), and bovine skin collagen (BC). The collagen composition was analyzed by SDS-PAGE, and the band profile of FC showed α1, α2, β, and γ chains without any degradation forms, resembling the patterns observed for RC, PC, and BC (Figure 1A). Next, we adjusted the collagen solution to a concentration of 1 mg/mL at a neutral pH to induce fibril formation. Notably, FC completed fibril formation within 5 min, a significantly faster process than RC, which took approximately 10 min. In contrast, BC and PC displayed slow and no fibril formation within the first 30 min, respectively (Figure 1B). This observed variation in fibril formation kinetics may be attributed to different collagen extraction methods, such as acidic, enzymatic, and alkaline extraction, which can influence the collagen structure and self-assembly differentially [14]. A complete fibril formation of BC or PC was only observed when collagen concentrations were increased to 2 or 3 mg/mL, respectively (Figures S1 and S2). Indeed, the intact molecular structure observed in FC is likely attributable to its manufacturing process involving acid solubilization.

### 2.2. The Effects of Tilapia Skin Collagen on Cell Adhesion, Proliferation, and Migration

**Figure 2:**
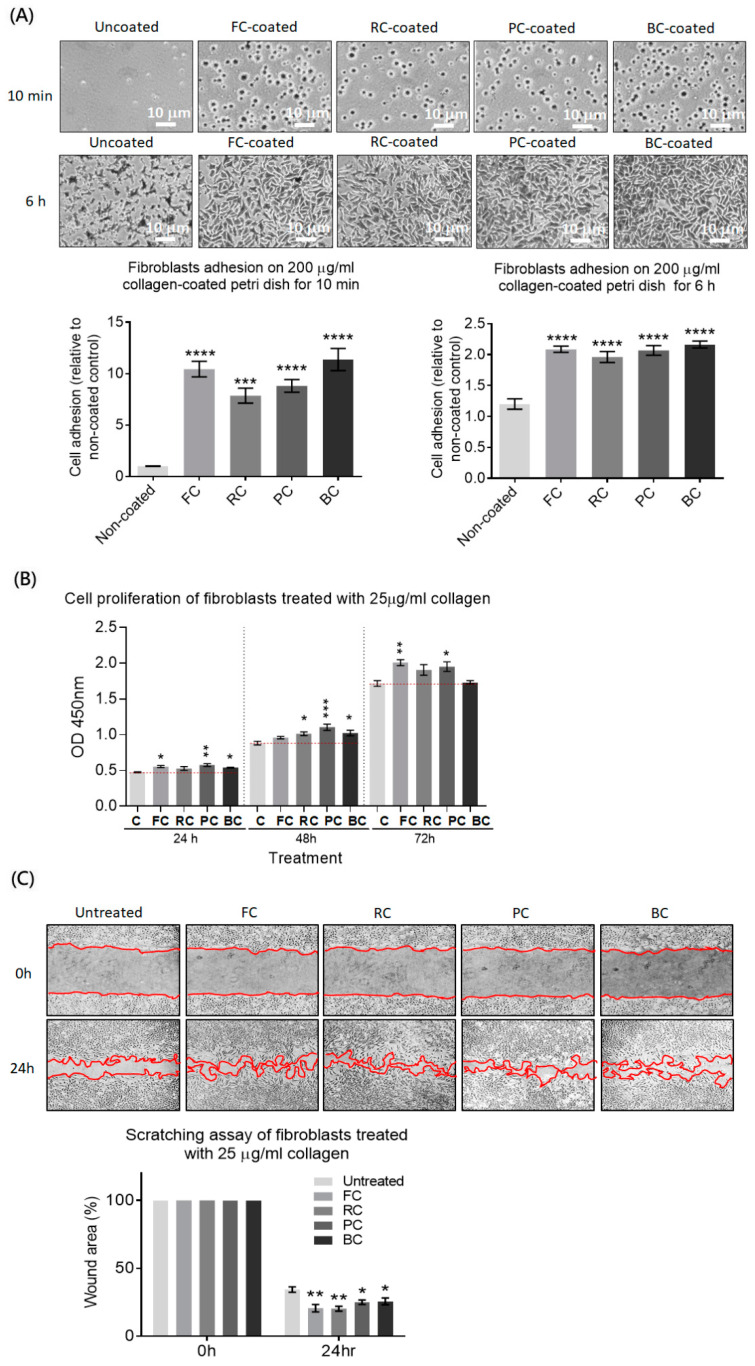
Evaluating the effects of the FC on cell adhesion, proliferation, and migration in a human skin fibroblast cell line compared to RC, PC, and BC. (**A**) The fibroblasts were seeded on the Petridishes coated with or without collagen (0.2 mg/mL). After washing gently, adherent cells were stained and counted under a microscope. The histogram shows the relative cell adhesion on the collagen-coated dish compared to the uncoated one. (**B**) The fibroblasts were seeded in a 96-well plate and treated with or without different collagen (25 μg/mL) for 24, 48, or 72 h. The supernatants were replaced with fresh medium containing CCK8 reagent to evaluate cell proliferation by measuring the absorbance at OD450 nm. (**C**) A cell monolayer of the fibroblasts was scratched and replaced with fresh medium containing different collagen (25 μg/mL) or no collagen. The scratched areas were recorded at 0 and 24 h and quantified using ImageJ 1.52q software. The percentage of wound area with or without the collagen treatment was shown in the histogram. Significance differences analyzed by one-way ANOVA were indicated as * *p* < 0.05; ** *p* < 0.01; *** *p* < 0.001; **** *p* < 0.0001.

Since type I collagen is the skin’s predominant component used to maintain the structure and provide physical anchorage sites for skin cells, we evaluated the effects of FC on the cell adhesion, proliferation, and migration of a human skin fibroblast cell line by comparing it to that of RC, PC, and BC. We observed that fibroblast adhesion was significantly enhanced on all four collagen-coated Petri dishes in a short incubation time of 10 min.Specifically, the average number of adherent cells on the FC-coated or BC-coated Petri dishes was similar and higher than that on the PC-coated or RC-coated dishes, although the difference was not statistically significant.After 6 h of incubation, we found that fibroblast adhesion covered the entire area of the observed field and spread well on all types of collagen-coated dishes. However, fibroblasts did not spread well and occupied a smaller area on the uncoated dish (Figure 2A).For cell proliferation, fibroblasts were treated with each type of collagen for 24, 48, or 72 h. We found that PC significantly enhanced cell proliferation at each time point. FC enhanced proliferation at 24 and 72 h, BCenhanced proliferation at 24 and 48 h, and RC onlyenhanced proliferation at 48 h. Thus, cell proliferation of fibroblasts was significantly upregulated in the presence of all four collagens at different time points. There was no significant difference among the different sources of collagens (Figure 2B).The scratching assay showed enhanced cell migration upon treatment with any four collagens. Notably, both FC and RC promoted more significant wound healing compared to PC or BC (Figure 2C). Taken together, these results indicate that the FC has a positive impact on cell adhesion, proliferation, and migration in the human skin fibroblast cell line, with similar or superior effects compared to other mammalian collagens.

### 2.3. Application of Tilapia Skin Collagen in Three-Dimensional (3D) Cell Culture

Three-dimensional cell culture is a promising method for generating organoids that could serve as a platform to study disease mechanisms or perform drug screening. Collagen can form hydrogels to provide a 3D environment. Given the fast fibril formation rate of FC and RC, we investigated their applicability in three different 3D cell culture models: the Madin–Darby Canine Kidney (MDCK) cell culture model for exploring the molecular mechanisms that regulate the renal epithelial cystogenesis, fibrogenesis, and tubulogenesis [15,16,17]; the fibroblast-induced gel contraction model for studying matrix contractions in wound healing, scar formation, and tissue remodeling [18,19,20]; and the interfacial invasiveness of a tumor spheroid model for investigating migrations and the invasion of cancers in matrix–substrate interfacial microenvironments [21,22,23]. The following results show that FC derived from tilapia skin holds promise as a valuable component in 3D cell culture systems.

#### 2.3.1. The MDCK 3D Culture Model

**Figure 3:**
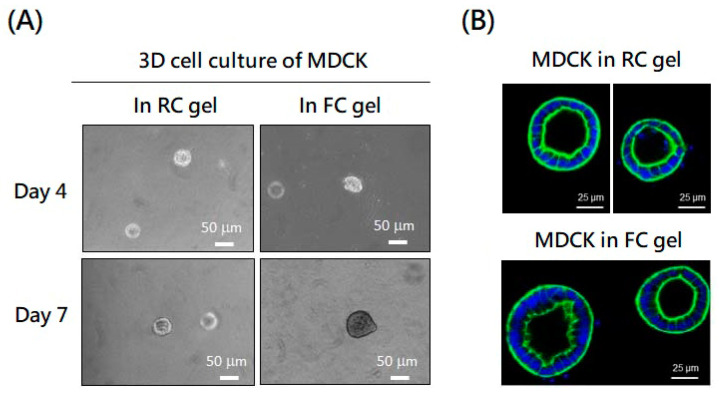
Application of fish collagen (FC) and rat collagen (RC) in MDCK three-dimensional culture. (**A**) MDCK cells (5000 cells/well) were suspended in FC or RC gel (1 mg/mL) at neutral pH in a 6-well plate to form a hydrogel. Spherical cysts derived from the MDCK embedded in both gels were observed and photographed on day 4 and day 7. (**B**) Sections of spherical cysts formed by MDCK in RC and FC gel were stained with DAPI (blue) and phalloidin dye (green) and observed using fluorescent microscopy.

Single MDCK cells were suspended in the FC or RC solution at a neutral pH to form a hydrogel. These cells embedded in both gels started to proliferate and organize into spherical cysts with similar forms and sizes (Figure 3A). The presence of hollow structures, consisting of a monolayer of epithelial cells surrounding a central lumen in RC and FC gel, was confirmed by immunofluorescent staining (Figure 3B). In addition, the average sizes of the cysts in the FC gel tended to be larger than in the RC gel (Supplementary Figure S3).This means that FC gel can support the proliferation and organization of MDCK cells into spherical cysts.

#### 2.3.2. The Fibroblast-Induced Gel Contraction Model

**Figure 4:**
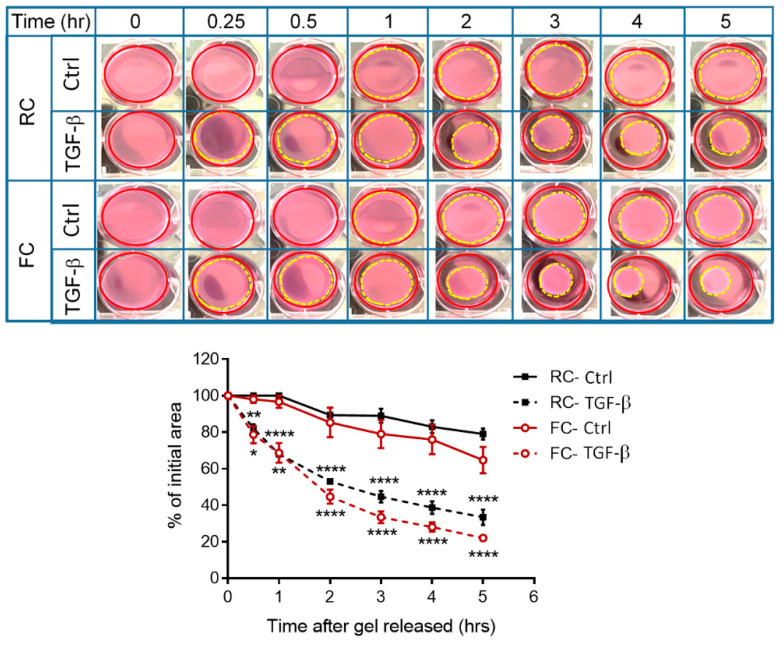
FC applied in the fibroblast-induced gel contraction model compared with RC. The fibroblasts (1 × 10^6^ cells/well) were suspended in FC or RC gel (1 mg/mL) at neutral pH in a 6-well plate to form a hydrogel. After 48 h incubation, the cells were treated with or without TGF-β, and the gels were released to initiate the contraction. Floating collagen gels were imaged at different time points. Changes in collagen gel size were measured using ImageJ software.The gel size changes in each well were calculated as follows: area of the contracted gel (yellow dash circle)/area of the well (red circle)× 100%. Significance differences analyzed bytwo-way ANOVAwere indicated as * *p* < 0.05; ** *p* < 0.01 and **** *p* < 0.0001 compared to control.

TGF-β is recognized for its ability to drive the differentiation of fibroblasts into myofibroblasts, a contractile and matrix-preserving cell type, which is pivotal in processes such as tissue repair, wound contractions, and fibrosis [24]. The fibroblasts embedded in the FC or RC gel were treated with or without TGF-β under the release of collagen gels from the sides of culture dishes. We found that the untreated fibroblasts exhibited a higher level of contraction in the FC gel compared to the RC gel. However, when treated with TGF-β, both collagen gels showed contractions at the same level (Figure 4). This means that FC gel is suitable for fibroblast-induced gel contractions with or without the presence of TGF-β.

#### 2.3.3. The Tumor Spheroid Interfacial Invasiveness Model

**Figure 5:**
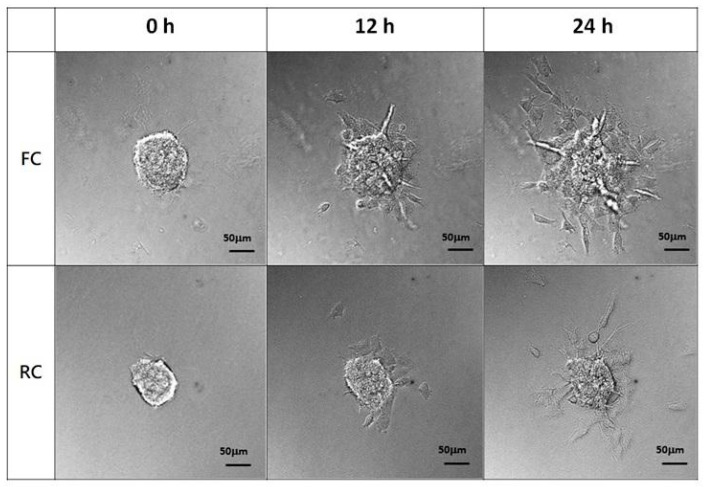
FC applied in the tumor spheroid interfacial invasiveness model compared with RC. MDA-MB-231 spheroids generated by culturing in ultra-low attachment plates were resuspended in neutral FC or RC solution and added toglass substrate for gelling. The spreading of the tumor spheroids on the glass was recorded by time-lapse microscopy for 24 h. The representative images of tumor spheroids at time points of 0 h, 12 h, and 24 h were shown.

The breast cancer cell MDA-MB-231 spheroids generated in an ultra-low attachment plate were resuspended in a neutral FC or RC solution and added onto the glass bottom dish for gelling. Via time-lapse microscopy, we observed the tumor spheroids in FC or RC gel spread radially on the glass substrate (Supplementary Videos S1 and S2). Interestingly, the tumor spheroid in FC gel exhibited a larger invasive area than that in RC gel (Figure 5). This means that FC gel can provide an environment to study 3D tumor spheroid interfacial invasions and even promote malignant behavior compared to RC gel.

### 2.4. The Effects of the Freeze-Dried FC Collagen Scaffold on Wound Healing

**Figure 6:**
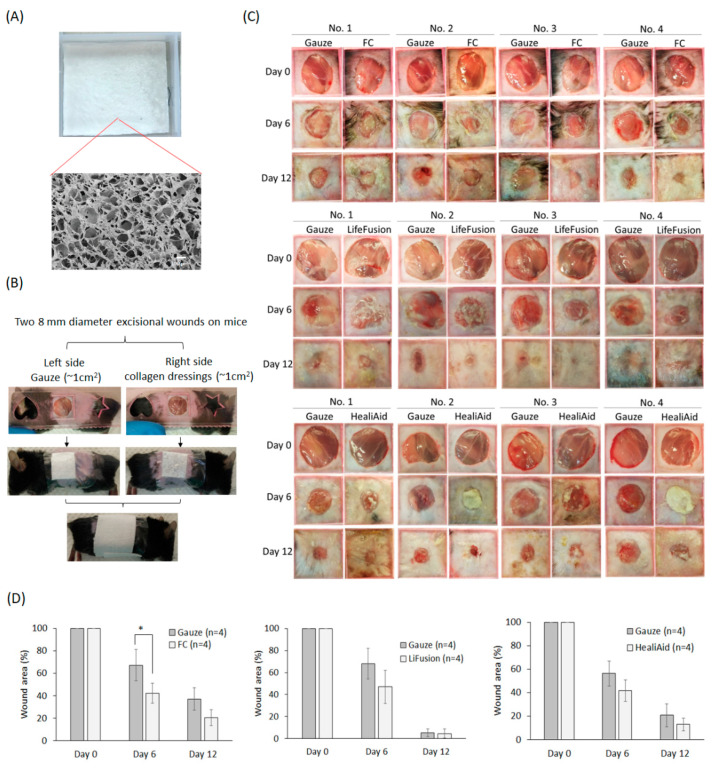
The effect of the tilapia skin collagen freeze-dried scaffold on excisional wound healing. (**A**) The fish skin collagen solution was freeze-dried in a mold, forming a collagen scaffold with a porous structure, which was observed by scanning electron microscopy (SEM). (**B**) Two 8 mm full-thickness excisional wounds were created on the same dorsal region of a mouse. The left-side wound was covered with gauze, while the right was covered with collagen dressings made from tilapia skin collagen (FC), LifeFusion, or HealiAid. The dressings were secured using Tegaderm and a bandage. On day 6, both the gauze and the collagen dressings were replaced. (**C**,**D**) The wound area was recorded using a digital camera on days 0, 6, and 12 and subsequently measured using ImageJ software. The Student’s *t*-test was employed to determine the statistical significance between the two groups. Statistical significance analyzed by Student’s *t*-test was indicated as * *p* < 0.05.

Collagen dressing promotes wound healing by facilitating the repair mechanisms in the wound bed. In this study, we prepared an FC freeze-dried scaffoldwith a homogeneous pore structure, as observed by scanning electron microscopy (SEM) (Figure 6A). To evaluate its effects, we used a mouse excisional wound model by creating two full-thickness wounds on the dorsal region. The right-side wound was covered with the FC collagen dressing, while the left-side wound was covered with a non-stick gauze (Figure 6B). The dressing was replaced once on day 6, and the wound area was recorded on days 6 and 12. For comparison, we also included two additional groups using commercial collagen dressings, the LifeFusion, made from porcine skin, and the HealiAid, made from bovine skin. The results showed that the average percentage of the wound area in these collagen-dressing-treated sides was reduced more than in the control-gauze-treated sides on both day 6 and day 12 in all three groups. However, only the FC scaffold group reached statistical significance on day six (Figure 6C). Interestingly, the fish skin collagen scaffold was quickly resolved on the wound bed within six days. This may be attributed to the scaffold not undergoing crosslinking treatment. In contrast, the LifeFusion and HealiAid collagen dressings remained intact on the wound site throughout the experiment period, and a few of the HealiAid dressings adhered to the wound and could not be removed. The result demonstrated that the FC freeze-dried scaffold significantly accelerates wound healing on day six post-injury, indicating its therapeutic potential compared to the current commercial product.We conducted H&E stain of tissue sections from wound samples collected on day 12.Our observations revealed that the appearances of hair follicles and the reteridges, small extensions protruding from the papillary dermis within the epidermis, were more pronounced in wounds repaired with FC, LifeFusion, or HealiAid dressings compared to those treated with gauze control. No significant difference between wounds treated with different collagen dressings (Supplementary Figure S4).

## 3. Discussion

Collagen is the most abundant extracellular matrix protein and has been widely used in 2D and 3D cultures to mimic the in vivo cellular environment, providing cell adhesion, proliferation, differentiation, and migration. Collagen used in research is mainly derived from mammalian tissues, which raises concerns about disease transmissionand religious restrictions. Fish has emerged as a safe alternative source of collagen for biomedical research. However, fish collagen products for research purposes are rare, and their biological functions remain largely unexamined. In this study, we examined commercial tilapia skin collagen and found that it promoted both cell adhesion and proliferation of human skin fibroblasts, similar to collagen from a rat tail, porcine skin, or bovine skin. Notably, the tilapia skin collagen, like rat tail collagen, showed a more significant stimulation of cell migration than that of porcine or bovine collagen. Furthermore, the tilapia skin collagen exhibited a fast fibril formation rate, implying its molecular structure is intact and able to form stable gels in a short time, making it suitable for 3D cell cultures. Indeed, tilapia skin collagen gel can provide a 3D environment for MDCK cell cyst formation, similar to rat tail collagen. However, the tilapia skin collagen gel exhibited higher sensitivity to fibroblast contractions and triggered a more pronounced interfacial invasion phenomenon of the breast cancer spheroid than the rat tail collagen gel. This could be due to differences in the physical and chemical properties between the two types of gels and requires further investigation.

Tilapia skin collagen exhibits higher thermal stability compared to collagen derived from cold-water fish, which displays values similar to that of mammalian collagen [25]. Type I collagen from rats, pigs, or bovines share a high protein sequence homology with human collagen, exceeding 90% [26]. However, the protein sequence similarity between fish and human collagen is approximately 80% [27]. Interestingly, the report indicated that the digestion forms of porcine skin collagen in human plasma after an oral injection were Pro-Hyp (95%), Leu-Hyp (3%), Ile-Hyp (1%), and Phe-Hyp (1%). In contrast, the digestion forms of fish skin collagen include Pro-Hyp (42%), Leu-Hyp (27%), Ala-Hyp (15%), Phe-Hyp (7%), Ile-Hyp (7%), and Pro-Hyp-Gly (3%) [28]. This suggests that different types or sources of collagen could lead to variations in detected peptides, which may have distinct biological activities. Fish skin collagen, with less homology to human collagen than mammalian collagen, may have alternative effects on cell activities that also require further investigation.

As a skin xenograft, the fish skin from North Atlantic cod, such as Kerecis^®^ Omega3 MariGen, was approved by the US Food and Drug Administration in 2013 to apply to burns and diabetic wounds [29,30]. Also, the tilapia fish skin xenografts have demonstrated good efficacy in treating full-thickness skin wounds in animals [31] and burn wounds in phase II and phase III randomized controlled trials, which led to complete wound reepithelialization, improved burn-related pain, and decreased treatment-related costs [32,33]. Moreover, a systematic review showed that fish skin collagen can improve the wound-healing rate and reepithelization in animal studies [34]. In this study, we generated the wound dressing by a freeze-drying FC solution into a scaffold without physical or chemical crosslinking. Since collagenase and gelatinase were upregulated in the wound site, the non-crosslinked FC scaffold applied on the wound site would be digested by the proteases [35]. We observed that the FC scaffold was quickly digested in the wound bed. Nevertheless, it significantly promoted the healing of the mouse’s excisional wounds. The collagen-degradation peptides have been demonstrated to serve as chemoattractants for fibroblasts in vitro, which might attract these cells to repair the damaged tissue [36]. Previous data have shown that collagen peptides derived from tilapia skin promoted cell migration and wound healing in rabbits [37]. In contrast, the commercial collagen dressings of the LifeFusion and HealiAid remained intact and easily stuck on the wound site during the experiment period, which might interfere with wound healing.

In conclusion, tilapia skin collagen, being economical and low-cost, would be an ideal product for biomedical research.

## 4. Materials and Methods

### 4.1. Cell Culture

The SV40T immortalized human skin fibroblast was obtained from AcceGenBiotechnology (ABI-TC301D, AcceGen, Fairfield, NJ, USA). The Madin–Darby Canine Kidney (MDCK) cell line and the breast cancer cell line MDA-MB-231 were purchased from the Bioresource Collection and Research Center (60004 and 60425, respectively, BCRC, Hsinchu, Taiwan). The fibroblastsand MDCK cells were cultured with low-glucose DMEM medium (31600034, Thermo Fisher Scientific, Waltham, MA, USA). These cells were cultured in a humidified incubator with 5% CO_2_ at 37 °C.

### 4.2. Collagen Solutions

Bovine collagen type I solution(10 mg/mL), referred to as BC, was obtained from Advanced Biomatrixcompany (#5133, Carlsbad, CA, USA). Rat tail collagen type I solution (3.65 mg/mL), referred to as RC, was purchased from Corning company (354236, Corning, NY, USA). Porcine collagen type I solution(4 mg/mL), referred to as PC, was obtained from SunmaxBiotechnology company (531-000-00, Tainan, Taiwan). Fish skin collagen type I solution(3.6 mg/mL), referred to as FC, was purchased from Innocollagene Biotechnology company (IB-CM001-100, Tainan, Taiwan).

### 4.3. SDS-PAGE

The composition of these commercial type I collagens was evaluated by SDS-PAGE. The collagen samples were mixed with a protein loading buffer and boiled for 10 min. The samples were separated by 5% and 7.5% resolving gel and stained with 0.5% Coomassie Blue R-250 (C.I. 42660, Sigma-Aldrich, Taufkirchen, Germany). The PageRuler™ Prestained Protein Ladder (26617, Thermo Fisher Scientific) was used to evaluate the molecular weights of the type I collagen.

### 4.4. Collagen Fibril Formation

Collagen can self-assemble to form fibrils at a physiological neutral pH in vitro. The in vitro fibril-forming ability was assessed in accordance with the procedures of R. A. Gelman et al. [38], with some modifications. Briefly, the concentration of the commercial type I collagen was adjusted to 3 mg/mL using 25 mM acetic acid. Then, 700 μL of collagen solution (3 mg/mL) was diluted with ddH_2_O to a total volume of 1800 μL and supplemented with 200 μL 10× PBS to neutralize the pH, resulting in a final concentration of around 1 mg/mL. The kinetic of collagen fibril formation was monitored at room temperature by measuring the absorbance value at 313 nm in a spectrophotometer based on turbidity change.

### 4.5. Cell Adhesion Assay

Petri dishes were coated with or without 0.2 mg/mL of the individual commercial type I collagen. The fibroblasts were seeded on the coated or uncoated Petri dishes (2 × 10^5^ cells/mL, 2 × 10^4^ cells/cm^2^) and incubated for 10 min following the protocol by Humphries, M. J. [39], with some modifications. Then, the Petri dishes were washed twice with PBS gently and stained with 0.25% crystal violet. The adherent cells were observed using microscopy and counted in five random fields (200×) by using a hand tally counter. The cell numbers from the coated Petri dishes were compared to thosefrom uncoated control.

### 4.6. Cell Proliferation Assay

The fibroblasts were seeded in a 96-well plate (5 × 10^3^ cells/well, 1.5 × 10^4^ cells/cm^2^) overnight. The cell culture supernatants were replaced with fresh medium containing individual commercial type I collagenat a concentration of 25μg/mL. The cells replaced with fresh medium served as the control. After 24, 48, or 72 h, the supernatant in each well was replaced with 100 μL fresh medium supplemented with 10 μL of CCK8 reagent. The plate was incubated for one hour at 37 °C. The absorbance of each well was measured at a wavelength of 450 nm. The proliferation rates were calculated by taking the difference in absorbance between the treated cells and the untreated control.

### 4.7. Cell Migration Assay

The fibroblasts were seeded in a 12-well plate (2.5 × 10^5^ cells/well, 7 × 10^4^ cells/cm^2^) overnight to reach confluency. The cell monolayer in each well was scratched in a straight line using a 1 mL pipette tip, which was held perpendicularly to the bottom of the well. The cell debris was removed by washing once with fresh medium, and fresh medium was added containing 25 μg/mL of individual commercial type I collagen. Fields of scratched lines were recorded by using a microscope with a digital camera at 0 h and 24 h time points. The scratched areas were quantified using ImageJ software. The percentage of wound healing was calculated according to the following formula: [(0 h scratched area − 24 h scratched area)/0 h scratched area] × 100%.

### 4.8. MDCK Cells in 3D Culture

Collagen gel (1 mg/mL) was prepared in accordance with the protocols of H.H. Lin et al. [15], with some modifications. In brief, 0.3 mL of the fish skin or rat tail collagen solution (3 mg/mL)was mixed with 0.1 mL of 5.7 × DMEM, 0.05 mL of 2.5% NaHCO_3_, 0.1 mL of 0.1 M HEPES, 0.01 mL of 0.17 M CaCl_2_, 0.01 mL of 1 N NaOH, and 0.43 mL of cell suspendedmedium (1 × DMEM) sequentially on ice. The mixtures were dispensed into a 6-well plate (1 mL/well) and placed immediately in a 37 °C incubator (5% CO_2_ in air, 37 °C) for at least 30 min to ensure complete gelation. After gelation, each culture was supplemented with 1.5 mL of culture medium. The spherical cyst formation was observed and photographed under an inverted microscope with a digital camera for 7 days. Finally, the spherical cysts containing hydrogel were rinsed with ice-cold PBS and fixed with 4% paraformaldehyde in PBS for 20 min at room temperature. Fixed cells were permeabilized with 0.5% Triton X-100 in PBS and blocked with SuperBlock buffer (Thermo Scientific, Rockford, IL, USA) for 1 h. After blocking, gels were stained with phalloidin-TRITC (Sigma-Aldrich) and 10 μg/mL DAPI and observed using confocal microscopy (FV-1000, Olympus, Tokyo, Japan).

### 4.9. Contractility Assay

Collagen contraction assays were prepared following the protocol by J.C. Howard et al. [40], with some modifications. Briefly, fibroblasts were embedded in a 3D collagen gel, as described in Section 4.8. The cell–collagen mixture was then aliquoted into a 12-well plate (1 mL/well) and immediately placed in a 37 °C incubator (5% CO_2_ in air, 37 °C) for at least 30 min to ensure complete gelation. After collagen polymerization, a medium with 1% FBS was added to the gel in each well. Following 24 h of incubation, some gels were treated with TGF-β (1 ng/mL) for an additional 24 h before the contraction assay. To initiate contraction, the collagen gels were gently released with a tip from the sides of the plate wells. The morphology of the collagen gels was captured at 0, 1, 2, 3, 4, and 5 h. The areas of contracted collagen gels and the original gels (the well size) in the images were measured using ImageJ software. Gel size changes in each well were calculated as follows: (area of the contracted gel/area of the original gel) × 100%.

### 4.10. Interfacial Invasiveness of Tumor Spheroid Assay

MDA-MB-231 cells were suspended in an ultra-low attachment 96-well round-bottom plate (7007, Corning) at a density of 3000 cells/well and incubated for 4 days to facilitate spheroid formation. The resulting spheroids were harvested by centrifugation at 500 rpm for 3 min in 15 mL centrifuge tubes at room temperature. Subsequently, the spheroids were resuspended in 50 μL of medium and mixed with 200 μL of a 2.5 mg/mL solution of either FC or RC (pH 7.4). The final concentration of the collagen in the mixture was 2 mg/mL similar to collagen densities in human fresh tissues [41]. The mixture was then applied onto an uncoated glass substrate and incubated at 37 °C for a minimum of 30 min until a solid gel formed. The morphological changes in tumor spheroid outgrowth were monitored using time-lapse differential interference contrast (DIC) microscopy over a period of 24 h.

### 4.11. Fabrication of Collagen Scaffold and Scanning Electron Microscopy Observation

The FC solution was freeze-dried and resolved with acid solution at 10 mg/mL concentration. The solution was poured into the mold with 6 cm (L) × 5 cm (W) and underwent the freeze-dried process to form the scaffold with 2 mm thickness. The FC scaffolds were subsequently disinfected with ozone for 20 min. The FC scaffold sample was also sent to the Instrument Center of National Cheng Kung University, Ministry of Science and Technology (MOST), for scanning electron microscopy observation using the ZEISS AURIGA equipment(Zeiss, Jena, Germany). At least 4 regions of different locations of the sample were imaged.

### 4.12. The Treatment of the Collagen Biomaterials on a Mouse Excisional Wound Model

The animal experiment was approved by the Institutional Animal Care and Use Committee of National Cheng Kung University (NCKU) (13 July 2020). Twelve C57BL/6 mice (male, 6-week-old) purchased from the Laboratory Animal Center, College of Medicine, NCKU, were maintained on standard laboratory food and water in the center. The mice were divided into three groups, including the LifeFusion porcine collagen dressing group (Life Fusion Inc., Tainan, Taiwan) (*n* = 4), the HealiAid bovine collagen dressing group (Maxigen Biotech Inc., Tainan, Taiwan) (*n* = 4), and the freeze-dried FC scaffold group (*n* = 4). After anesthetizing the shaved mice, two full-thickness wounds were made using an 8 mm skin biopsy punch on the left and right sides of the mouse dorsal region. The left wound was covered with 1 cm^2^ non-stick gauze (Absorbent Nonwoven Pad, 3M), and the right-side wounds were covered with 1 cm^2^ different collagen dressing. Then, the gauze or collagen dressings were fixed on the skin withthe adhesive Tegaderm^TM^ (3M) and a breathable bandage. These gauze or collagen dressings were renewed on day 6. The wound area was aligned in a hollow square (1 cm × 1 cm) of a ruler and photographed by a digital camera on days 6 and 12. The wound and the hollow square areas were measured using the ImageJ software (NIH). The wound area was normalized by the hollow square area. Then, the percentage of the normalized wound area was calculated as follows: (normalized wound area of day 6 or day 12/normalized wound area of Day 0) × 100. The wound samples of each group were collected on day 12 to make formalin-fixed and paraffin-embedded tissue sections. The sections were stained with H&Eto analyze the wound repair.

### 4.13. Data Analysis

The experiment data were performed in triplicate and expressed as the mean ± standard deviation (SD). The differences between the test and control groups were evaluated using ANOVA or Student’s *t*-test in the GraphPad Prism 5 software (GraphPad Software Incorporation, San Diego, CA, USA). Significant differences were set as * *p* < 0.05.

## Data Availability

Data are contained within the article and the Supplementary Materials.

## Supplementary Materials

The following supporting information can be downloaded at https://www.mdpi.com/article/10.3390/molecules29020402/s1: Figure S1. The fibril formation kinetics of bovine collagen. The bovine collagen was treated with H_2_O and 10X PBS to achieve a neutral solution with a concentration of 1 or 2 mg/mL. The kinetics of fibril formation was assessed by measuring the optical density (OD) at 313 nm over a 60-min period using a spectrophotometer; Figure S2. The fibril formation kinetics of porcine collagen. The porcine collagen was treated with H_2_O and 10X PBS to achieve a neutral solution with a concentration of 1, 2, or 3 mg/mL. The kinetics of fibril formation was assessed by measuring the optical density (OD) at 313 nm over a 60-min period using a spectrophotometer; Figure S3. The size of MDCK cysts in FC and RC gel. (A) The random fields of MDCK cysts in RC or FC gel on day 4 and day 7 under microscopy observation. The sizes of the cysts in the gel were calculated according to the scale bar. (B) The histogram of the cysts particle in RC and FC gel was shown. The average sizes of the cysts in FC gel tended to be larger than that in RC gel; Figure S4. H&E stain of the tissue sections of wound samples. The wound samples of each group was collected on day 12 for making formalin-fixed and paraffin-embedded tissue section. The sections were stained with H&E for analyzing the wound repair. The representative picture of the H&E stain of each group was shown. The hair follicles (red arrow head) and the Rete-ridges (red arrow), small extensions protruding from the papillary dermis within the epidermis, were point out in the repair wounds of FC (A), LifeFusion (B) and HealiAid (C) groups. Video S1. The interfacial invasion of MDA-MB-231 breast cancer cell spheroid in FC gel; Video S2. The interfacial invasion of MDA-MB-231 breast cancer cell spheroid in RC gel.
