# Supplementary material for: Assessment of Tilapia Skin Collagen for Biomedical Research Applications in Comparison with Mammalian Collagen
Source: Molecules. 2024 Jan 13;29(2):402. doi: 10.3390/molecules29020402 (PMC10819363; doi:10.3390/molecules29020402)
Supplement: Supplementary file 1 [file molecules-29-00402-s001.zip › Supplementary figures and legends.pdf]

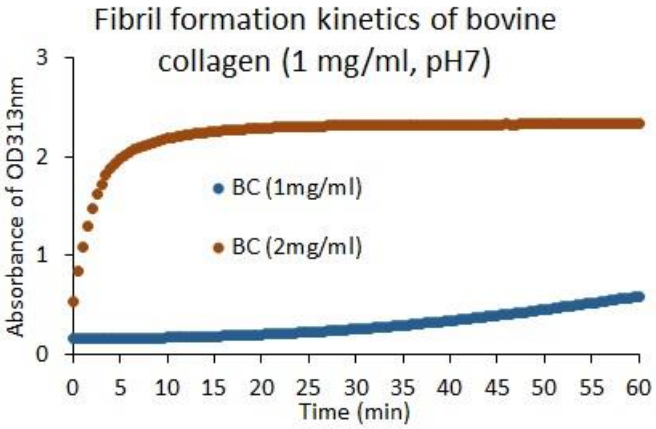

Supplementary Figure S1. The fibril formation kinetics of bovine collagen. The bovine collagen was treated with H_2O and 10X PBS to achieve a neutral solution with a concentration of 1 or 2 mg/ml. The kinetics of fibril formation was assessed by measuring the optical density (OD) at 313 nm over a 60-min period using a spectrophotometer.

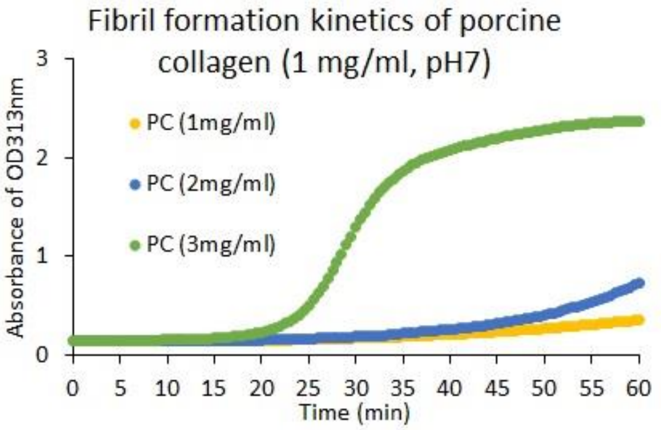

Supplementary Figure S2. The fibril formation kinetics of porcine collagen. The porcine collagen was treated with H_2O and 10X PBS to achieve a neutral solution with a concentration of 1, 2, or 3 mg/ml. The kinetics of fibril formation was assessed by measuring the optical density (OD) at 313 nm over a 60-min period using a spectrophotometer.

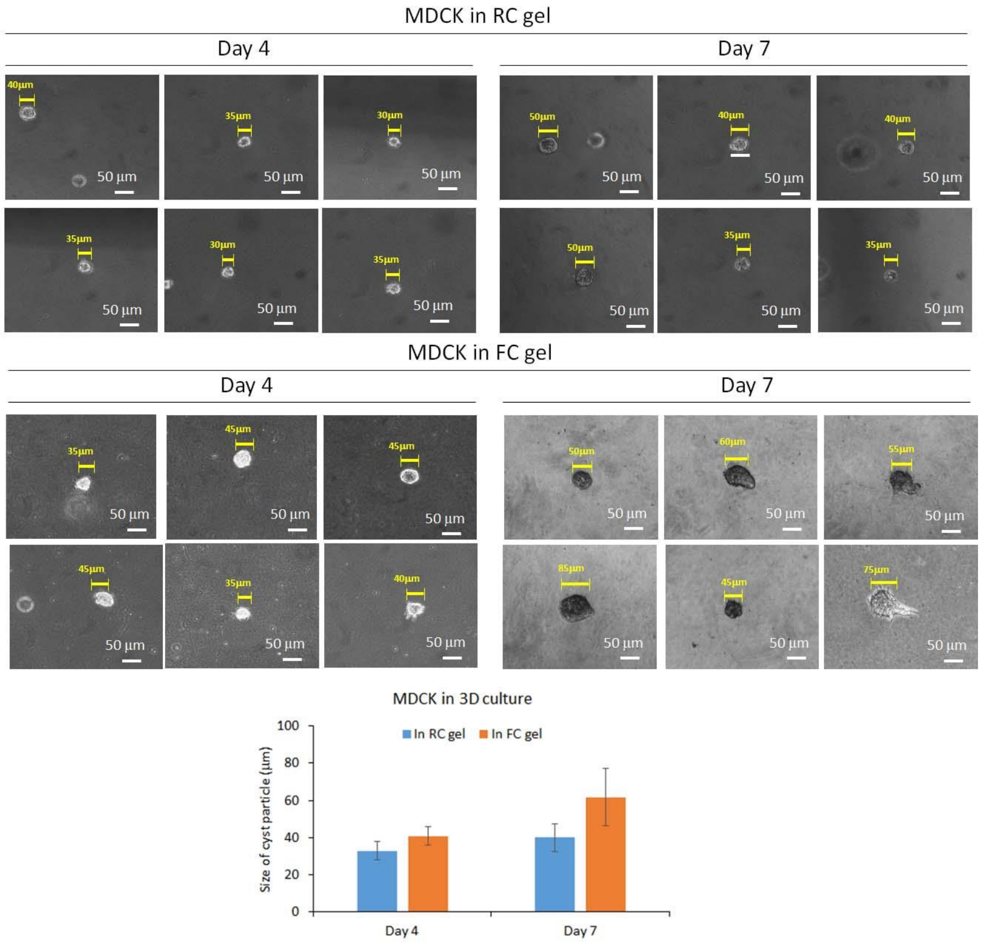

Supplementary Figure S3. The size of MDCK cysts in FC and RC gel. (A) The random fields of MDCK cysts in RC or FC gel on day 4 and day 7 under microscopy observation. The sizes of the cysts in the gel were calculated according to the scale bar. (B) The histogram of the cysts particle in RC and FC gel was shown. The average sizes of the cysts in FC gel tended to be larger than that in RC gel.

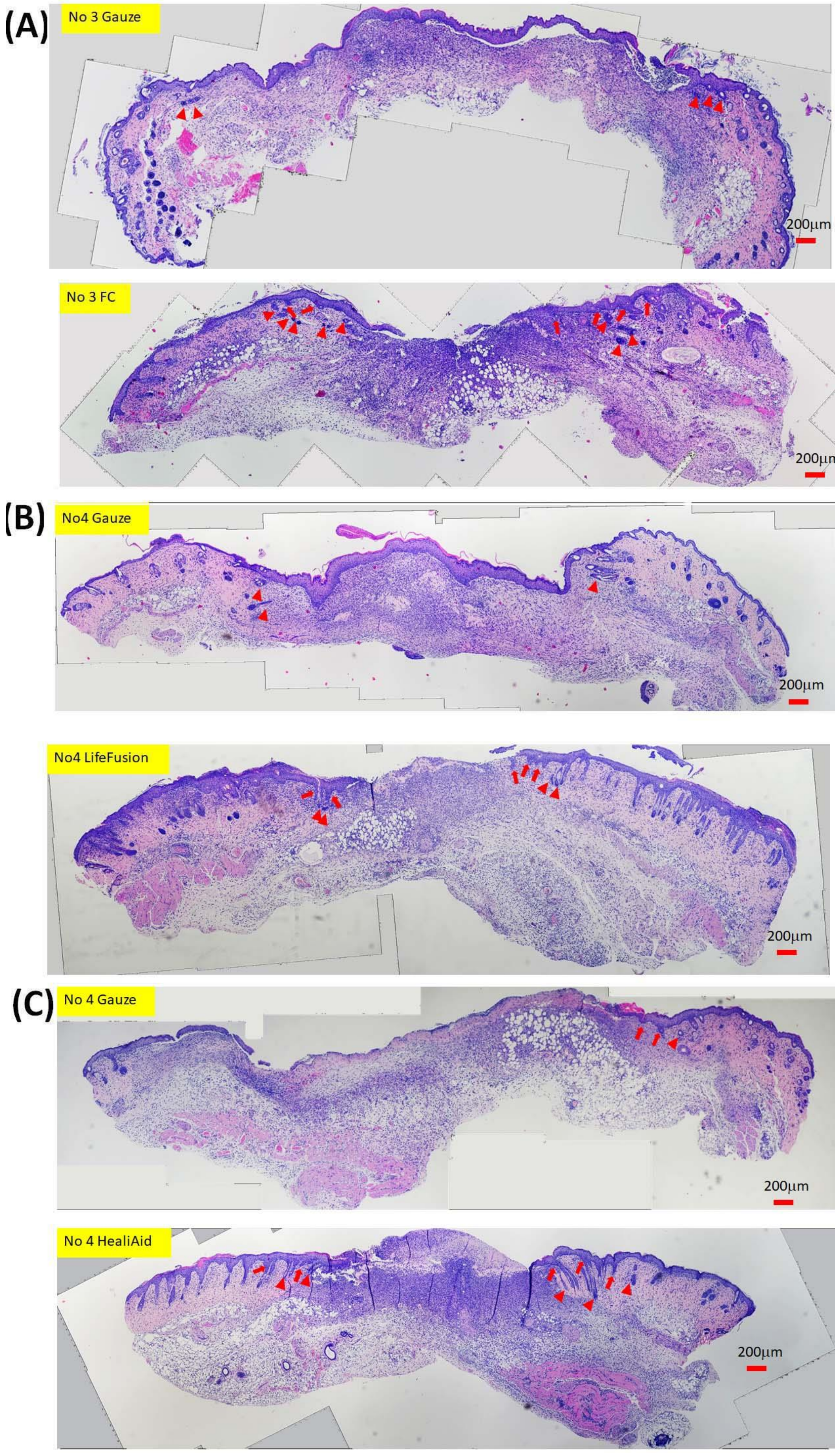
(A)
No 3 Gauze
200μm
No 3 FC
200μm
(B)
No4 Gauze
200μm
No4 LifeFusion
200μm
(C)
No 4 Gauze
200μm
No 4 HealiAid
200μm

Supplementary Figure S4. H&E stain of the tissue sections of wound samples. The wound samples of each group was collected on day 12 for making formalin-fixed and paraffin-embedded tissue section. The sections were stained with H&E for analyzing the wound repair. The representative picture of the H&E stain of each group was shown. The hair follicles (red arrow head) and the Rete-ridges (red arrow), small extensions protruding from the papillary dermis within the epidermis, were point out in the repair wounds of FC (A), LifeFusion (B) and HealiAid (C) groups.
